# Correction: SABRE-SHEATH hyperpolarized ^15^N_2_-imidazole for Zn^2+^ sensing

**DOI:** 10.1039/d6cc90108e

**Published:** 2026-04-02

**Authors:** Brojo Kishor Shachib Dhali, Abubakar Abdurraheem, Mustapha Abdulmojeed, Anna Samoilenko, Megan Pike, Rielly J. Harrison, Franziska Theiss, Boyd M. Goodson, Eduard Y. Chekmenev, Thomas Theis

**Affiliations:** a Department of Chemistry, North Carolina State University Raleigh North Carolina 27965-8204 USA bdhali@ncsu.edu ttheis@ncsu.edu; b Department of Chemistry, Karmanos Cancer Institute, Wayne State University Detroit MI 8202 USA gq3501@wayne.edu; c School of Chemical & Biomolecular Sciences, Southern Illinois University Carbondale Illinois 62901 USA

## Abstract

Correction for ‘SABRE-SHEATH hyperpolarized ^15^N_2_-imidazole for Zn^2+^ sensing’ by Brojo Kishor Shachib Dhali *et al.*, *Chem. Commun.*, 2025, **61**, 12115–12118, https://doi.org/10.1039/D5CC02890F.

Due to communications received from the National Institutes of Health that specify that a subject matter expert concluded it was unlikely that the 2024 *ACS Sensors* publication^1^ was a product of the R21EB033872 award, the co-authors should not have acknowledged the NIH grant R21EB033872 for this publication by Dr Chekmenev. Based on the arguments presented by the subject matter expert, this *ChemComm* publication was unlikely to be a product of the R21EB033872 award.

The Royal Society of Chemistry apologises for these errors and any consequent inconvenience to authors and readers.

## References

[cit1] Nantogma S., de Maissin H., Adelabu I., Abdurraheem A., Nelson C., Chukanov N. V., Salnikov O. G., Koptyug I. V., Lehmkuhl S., Schmidt A. B., Appelt S., Theis T., Chekmenev E. Y. (2024). ACS Sens..

